# Evaluation of the Comprehensive Complication Index Versus the Clavien–Dindo Classification for Predicting Clinical Outcomes After Cardiac Surgery in Adult Patients

**DOI:** 10.3390/jcdd12120461

**Published:** 2025-11-27

**Authors:** Xinfang Zhang, Lu Zhang, Jimei Chen, Huigen Huang, Huan Ma, Jinlin Wu, Shuyuan Tan, Xiangyu Cai, Hongru Zhu, Ling Wang

**Affiliations:** 1 Faculty of Medicine, Macau University of Science and Technology, Macau SAR, China; 3230006370@student.must.edu.mo (X.Z.);; 2 Guangdong Provincial People’s Hospital (Guangdong Academy of Medical Sciences), Southern Medical University, Guangzhou 510080, China

**Keywords:** Clavien–Dindo classification, comprehensive complication index, cardiac surgery, postoperative complication, clinical outcome

## Abstract

Background: Adult patients undergoing cardiac surgery are at an elevated risk of experiencing postoperative complications. However, there is currently no consensus on the most accurate instrument for assessing clinical outcomes following the occurrence of such complications in cardiac surgery. Objective: The objective was to validate the comprehensive complication index (CCI^®^) and Clavien–Dindo classification (CDC) regarding their ability to evaluate clinical outcomes in adult cardiac surgery. Methods: This retrospective study included 1896 adult patients who underwent cardiac surgery between September 2023 and October 2024. Among these patients, 849 developed postoperative complications. Complications were graded using the CDC, which were then converted to the CCI^®^. The validation of the CCI and CDC was evaluated. The strength of the correlation between the CCI^®^/CDC and clinical outcomes, including ICU stay duration, length of hospital stay, and hospitalization cost were compared using Spearman’s ρ and Fisher’s z-transformation. We also employed generalized linear models to analyze the variables that influenced clinical outcomes. Results: The median age of the patients was 58.0 years; the median CCI^®^ score was 0.0 (interquartile range [IQR]: 0.0, 20.9). Pneumonia (92.8%) was the most common complication. The correlation of the CCI^®^ with postoperative outcomes was stronger than the CDC: ICU stay (ρ = 0.786 vs. 0.401, *p* < 0.001), LOS (ρ = 0.465 vs. 0.342, *p* = 0.002), and hospitalization cost (ρ = 0.602 vs. 0.354, *p* < 0.001). Conclusions: Both the CCI^®^ and CDC are valid tools for evaluating postoperative outcomes, while the CCI^®^ has superior discriminative ability for evaluation ICU stay duration, LOS, and hospitalization cost in adult cardiac surgery patients.

## 1. Introduction

Postoperative complications after major surgery are common and lead to an increase in the length of the hospital stay and the medical expenditure [1]; cardiac surgery faces more postoperative complications than other general surgeries [2]. Cardiopulmonary bypass (CPB) is a widely used procedure during cardiac surgeries. During the CPB process, ischemic injury to the heart caused by aortic cross clamping (ACC), followed by reperfusion injury after the clamp release, results in alterations to the tissue microenvironment, including pH dysregulation, accumulation of free radicals, and cell death [3,4]. The literature indicates that the patients undergoing cardiac surgeries presented more comorbidities and reduced physiological reserve, as well as age-related frailty in elderly patients [5,6].

The EuroSCORE II is widely used to predict the risk of death in patients after cardiac surgeries; however, it has inherent limitations, particularly in elderly patients, and is less effective in predicting postoperative complications [7]. The calculator currently used by the Society of Thoracic Surgeons (STS) predicts postoperative mortality and morbidity. However, it cannot provide a risk stratification of the postoperative outcome for ICU stays [8]. There is currently no universally accepted standard for evaluating the clinical outcome after cardiac surgery.

The Clavien–Dindo classification (CDC) represents the most widely utilized grading system for assessing complications across various surgical specialties [9]. It consists of a five-grade scale based on the invasiveness of the treatment required to manage complications, ranging from the mildest (Grade I) to the most severe (Grade V), with Grade V indicating death. Postoperative complications are categorized as mild or severe based on the CDC, with Grade III serving as the threshold for differentiation.

An extension of this grading system is the comprehensive complication index (CCI^®^) [10,11]. The CCI^®^ captures the overall morbidity associated with postoperative complications by summarizing both the number and severity of complications. It provides a continuous scale ranging from 0 (no complication) to 100 (death). Although the CCI^®^ has been validated and widely accepted as a standard classification system for complications in general surgery [12,13], its use in adult cardiac surgery remains limited.

The study aimed to evaluate the validation of two commonly used instruments, the CDC and CCI^®^ in evaluating clinical outcomes after cardiac surgery: the length of ICU stays, length of hospital stays (LOS), and hospitalization cost among adult patients. We hypothesized that the adult patients undergoing cardiac surgeries may face an increased likelihood of experiencing multiple and severe complications; hence, the use of the CCI^®^ could offer a more accurate evaluation of the postoperative outcomes compared with the CDC. Furthermore, we posited that the risk factors influencing postoperative outcomes would be multifaceted.

## 2. Materials and Methods

### 2.1. Design and Subjects

This is a retrospective study based on the medical records and the article is reported according to the STROBE guidelines (Supplementary file STROBE checklist). The subjects were the adult patients scheduled for cardiac surgery from September 2023 to October 2024 at a tertiary hospital located in Guangzhou, China. The inclusion criteria were as follows: (1) age ≥ 18 years and (2) had received cardiac surgeries. The exclusion criteria were as follows: (1) had combined surgeries involved with other organs at the same time; (2) had cardiac surgeries without general anesthesia; and (3) the decision to be discharged from the hospital was not made by the physician, but the patients or their family members instead. 

### 2.2. The Parameters and Data Collection

The general data were extracted or calculated from the hospital information system (HIS). The following data were collected: gender; age; body mass index (BMI); type of residence; marital status; comorbidity with other diseases; resuscitation records; type of cardiac surgeries; total CCI^®^ scores, as well as CCI^®^ scores for mild and severe complications; patient distribution across different grades of CDCs; incidence of single and complex postoperative complications; whether having the procedure of cardiopulmonary bypass (CPB) during cardiac surgery; utilization of albumin or blood products during the process of CPB; duration of operation, CPB, and aortic cross clamp (ACC); ICU stay; LOS; and hospitalization cost (Chinese Yuan, CNY).

The assessment of postoperative complications was conducted utilizing both the CDC and CCI^®^. The CDC categorizes complications into five grades, ranging from grade I to grade V in terms of severity, with grade I representing the least severe and grade V indicating death as the most severe outcome. Grade I includes any deviation from the normal postoperative course without the need for pharmacological treatment or surgical, endoscopic, and radiological interventions. Accepted therapeutic regimens for the cases are as follows: drugs as antiemetics, antipyretics, analgesics, diuretics and electrolytes, and physiotherapy. This grade also includes wound infections opened at the bedside. Grade II encompasses those requiring drug treatment and containing blood transfusions and total parenteral nutrition. Grade III includes those requiring surgical, endoscopic, or radiological intervention. Grade III are divided into IIIa and IIIb according to whether the intervention is implemented under general anesthesia (IIIb) or not (IIIa). Grade IV includes life-threatening complications that require intermediate care or ICU management. Grade IV is divided into IVa and IVb according to whether the complication is single or multiple organ dysfunction.

The CCI^®^ integrates all recorded complications and weighs their severity according to the CDC [10]. The CCI^®^ calculator formula is available from the website https://www.cci-calculator.com/cciCalculator (accessed on 20 November 2024).

### 2.3. Data Analysis

Categorical variables were presented as frequency and percentage, continuous variables were presented as mean ± standard deviation (SD) or median and interquartile range (IQR) according to the data distribution pattern.

Univariable linear regression, lasso regression, and generalized linear models analyses were performed to identify the risk factors for ICU stay, LOS, and hospitalization cost among the patients who received cardiac operation. The variables that demonstrated significant results (*p* < 0.05) in the univariable linear analysis were subsequently included in the lasso regression analysis. Correlation coefficients of both CCI^®^ and CDC for ICU stay (day), LOS (day), and hospitalization cost (CNY) were calculated using Spearman’s ρ tests and compared using standard Fisher’s z-transformation [14]. The strength of correlation coefficients was categorized as weak (0.10 to 0.29), moderate (0.30 to 0.49), and strong (0.50 to 1.00) [15]. All analyses were performed using SPSS (version 27, Chicago, IL, USA) and R 4.5.2, *p* < 0.05, indicated a significant difference.

## 3. Results

### 3.1. General Information of the Subjects

A total of 1942 subjects were screened who underwent cardiac surgeries between September 2023 and October 2024, primarily. After eliminating 46 cases (8 having duplicated calculations, 36 patients re-hospitalized for complications, and 2 self-discharged), a final total of 1896 subjects were included in the study; among them, 849 patients developed complications. The median (interquartile range, IQR) age was 58.0 years (49.0, 65.0), 1121 (59.1%) were males, and 953 (50.3%) patients had no comorbidities. Hypertension was the most common comorbidity, accounting for 20.1%, other comorbidities were diabetes, hypertension with diabetes, stroke, renal dysfunction, gastrointestinal disease, etc. The details are shown in Table 1. The number of patients who underwent valve surgery was 1115 (58.8%), making it the most common type of cardiac surgery, followed by aortic dissection (AD) surgery, which accounted for 282 cases (14.9%). There were 223 (11.8%) patients who received albumin and 223 (11.8%) received blood products during the process of CPB. Among the 849 patients with postoperative complications, 124 had resuscitation records.

The overall median CCI^®^ score was 0.0 (0.0.20.9), while the median scores for mild and severe complications were 20.9 (20.9, 29.6) and 45.6 (37.1, 58.0), respectively. The Grade II accounted for the highest proportion, with 635 cases (33.5%), according to the CDC. Median surgical time was 340 (289, 410) minutes, median CPB and ACC time were 147 (108, 192) minutes and 86 (57, 115) minutes, respectively.

There were 22 postoperative complications that occurred in the study population, with pneumonia being the most prevalent, accounting for 788 cases (92.8%) of the 849 patients with complications. The maximum number of complications observed in a single patient was 10. The quantity and percentage of the postoperative complications in the patients are shown in Table 2. The information for severity and intervention of postoperative complications is shown in the Supplementary Material.

### 3.2. Risk Factors for Clinical Outcomes

The univariable linear regression was used to test the association of different demographic or biochemical variables with the following clinical outcomes: ICU stay, LOS, hospitalization cost. We incorporated variables with significant results (*p* < 0.05) from univariable analysis into lasso regression analysis. Additionally, we employed generalized linear models to identify the risk factors associated with clinical outcomes. Senior age, history of resuscitation, prolonged surgery time, albumin supplement, elevated CCI^®^ score and higher BMI were identified as the risk factors for ICU stay; other types of surgeries were protective factors for ICU stay. Senior age, history of resuscitation, prolonged surgery time, elevated CCI^®^ score, having comorbidities before surgery, and CABG surgery were the risk factors for LOS. Senior age, history of resuscitation, prolonged surgery time, albumin use, and higher CCI^®^ score were the risk variables for hospitalization cost; CABG and other types of surgeries emerged as protective factors for hospitalization cost. (Table 3)

### 3.3. Evaluation of the CCI^®^ and CDC on Clinical Outcomes for the Subjects

**Figure 1:**
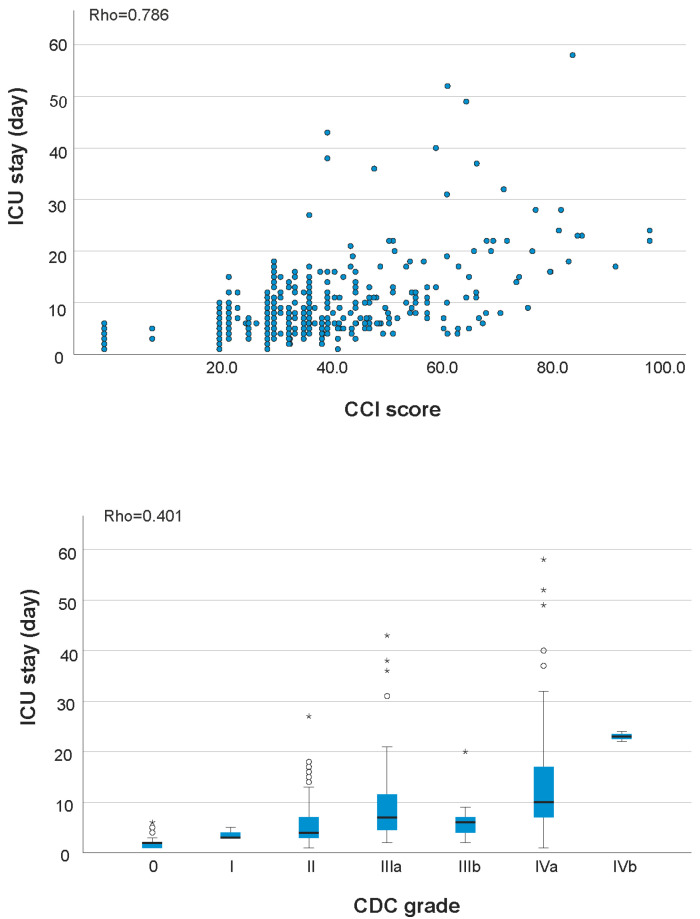
CCI^®^ score (**top**) and CDC grade (**bottom**) versus ICU stay of the subjects.

**Figure 2:**
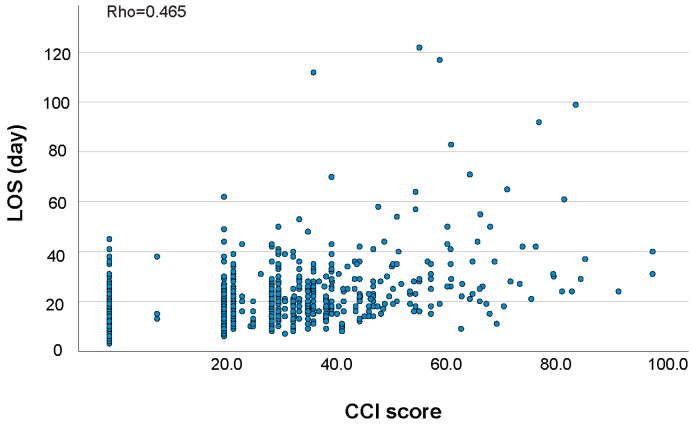

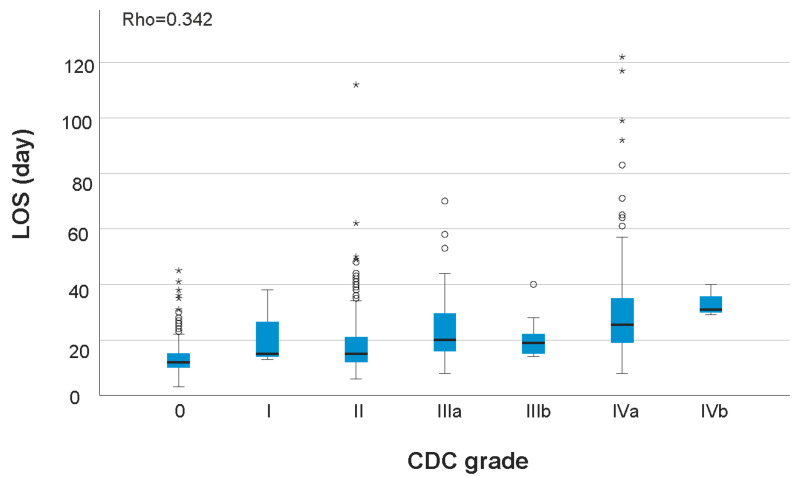
CCI^®^ score (**top**) and CDC grade (**bottom**) versus LOS of the subjects.

**Figure 3:**
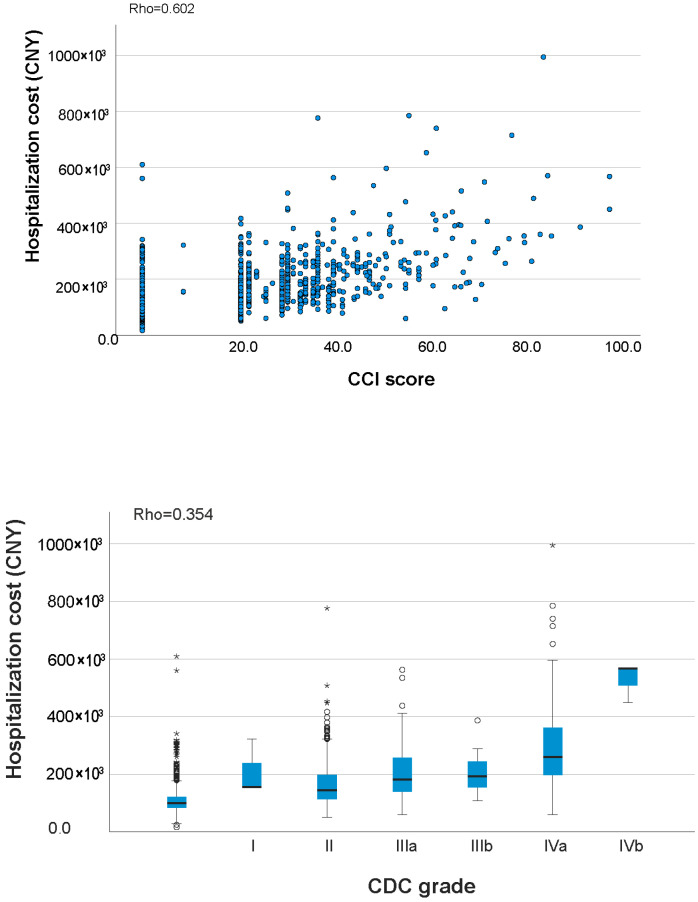
CCI^®^ score (**top**) and CDC grade (**bottom**) versus hospitalization cost of the subjects.

A strong correlation was found between the CCI^®^ and CDC (ρ = 0.715, *p* < 0.001). Both CCI^®^ and CDC demonstrated significantly strong or moderate correlation with ICU stay (ρ = 0.786 vs. 0.401, *p* < 0.001); LOS (ρ = 0.465 vs. 0.342, *p* = 0.002); and hospitalization cost (ρ = 0.602 vs. 0.354, *p* < 0.001) as shown in Table 4. The scatterplot of CCI^®^ and CDC for ICU stay, LOS, and hospitalization cost are shown in Figure 1, Figure 2 and Figure 3.

## 4. Discussion

Based on our findings, both the CCI^®^ and CDC demonstrated a strong capacity to assess the severity of complications in the patients after cardiac operation, while the CCI^®^ exhibited a stronger correlation with the clinical outcomes of the ICU stay, LOS, and hospitalization cost compared with the CDC.

The CCI^®^ represents the overall magnitude of all complications. Continuous monitoring of the CCI^®^ could indirectly reflect the surgical performance [16]. In our study, 22 postoperative complications were reported in 849 subjects. The diverse complications of post operation in these cardiac patients may have multifactorial etiologies, including the systemic inflammatory response (SIRS) induced by CPB [17,18], the prolonged surgical and CPB duration [19,20], the complexity of surgical procedures [21,22], and existing risk factors, such as senior age and multiple comorbidities [23,24]. The type and severity of postoperative complications may differ between studies and treatment [25]; the heterogeneity shows the need for standardized reporting for complications in adult cardiac surgery.

The CCI^®^ evaluates each complication and generates a comprehensive score that encompasses almost all complications. It can show a sequential progression to serious outcomes [26]. The severe complication rate was 24.8% among all the complication samples, which is higher than the incidence in general surgery populations [27]. The median CCI^®^ score for severe complications in adult cardiac surgery patients was 45.6 in this study, exceeding the CCI^®^ cutoff value for liver cancer surgery [2] and advanced ovarian cancer surgery [28].

The ICU stay and LOS can be regarded as major markers for clinical outcomes following surgery. In this study, both CCI^®^ and CDC demonstrated significant correlations with ICU stay and LOS, indicating that both instruments are valid for evaluating these parameters.

Triemstra claimed that both the CCI^®^ and CDC showed weak correlations with ICU stay in patients who received a D2-gastrectomy; however, the author did not report the median duration of the ICU stay [29]. In our study, the median duration of ICU stay was two days (1.0. 4.0). The CDC identifies and categorizes only the most severe complications; however, the actual burden of complications—particularly in the patients with collateral issues—may be underestimated, potentially resulting in data loss [28]. In our research, 487 patients (25.7%) experienced more than one postoperative complication. The CCI^®^ assessed each complication and weighed it with the appropriate scores, thereby enhancing its discriminative power in predicting the duration of the ICU stay of the patients. Thus, the CCI^®^ has the potential to more precisely evaluate the duration of ICU stay duration than the CDC.

Previous research indicated that the CCI^®^ was more strongly correlated with the LOS than the CDC in the patients undergoing pelvic exenteration [30], colorectal resections [31], and pancreatic surgical procedures [32]. The findings of our study were consistent with these reports. When comparing the indicator of LOS, the CCI^®^ demonstrated superior discriminative performance than the CDC in our research.

Hospitalization cost offers another marker to evaluate clinical outcomes and postoperative complications. The CCI^®^ was deemed valid regarding the overall postoperative cost for general surgeries [33]. A prospective study revealed a strong correlation between postoperative complications and the total cost of medical care for abdominal surgeries [11]. Smeyers reported that the CCI^®^ exhibited stronger association with the healthcare cost after colorectal resections [31], which aligns with our research findings. Danilovic showed that the CDC underestimated the medical expenditure during hospitalization for the patients who underwent percutaneous nephrolithotomy in comparison with the CCI^®^ [34]. In our research, both the CCI^®^ and CDC were valid metrics for assessing the healthcare burden in the case of postoperative complications in cardiac disease patients, while the CCI^®^ was superior to the CDC in evaluating the hospitalization cost. An elevated CCI^®^ score was identified as a risk factor for a longer ICU stay, LOS, and increased hospitalization cost through the analysis using generalized linear models. An elevated CCI^®^ score implies severe and/or complicated complications, which contribute to greater medical expenditure during patient hospitalization. Cai reported that there were no significant differences between the two instruments and total hospitalization cost, because of different types of medical insurance [35]. We utilized the total medical expenses incurred during the entire hospitalization period, excluding any deduction from medical insurance. This methodology served to mitigate potential biases related to medical insurance payment in our findings.

Our research highlights several strengths and innovations. Cardiac surgery is an inherently complex procedure, associated with numerous postoperative complications. This study applied scientific and practical measurement tools for assessing the risk of complications following cardiac surgery. As previously mentioned, both the EuroSCORE II and STS are widely utilized tools for predicting the potential risks and severity associated with patients undergoing cardiac surgeries. However, there is a relative scarcity of metrics available to assess the severity of postoperative complications that occurred following these procedures. Our research enhances the existing body of knowledge on postoperative complications in cardiac surgeries and provides quantitative measurements for evaluating the severity of such complications after cardiac procedures. This research demonstrated that the CCI^®^ can serve as a precise predictor to identify high-risk patients undergoing cardiac surgeries, so that cardiac surgeons, rehabilitation therapists, and nurses can take prophylactic measures in time to prevent or mitigate severe complications.

This study had some limitations. First, complications were only recorded during hospitalization; future research should evaluate the effectiveness of the CCI^®^ in predicting post-discharge complications. Second, we did not incorporate the information regarding frailty, which had gained increased attention regarding perioperative risk and clinical outcomes; this omission might cause bias in the results [36]. Third, the study was implemented in a single cardiac center and did not include death cases, limiting its generalizability to high-risk populations. Potential influencing factors, such as disparities in surgical technique, hospital medical resources, and management levels might cause bias in the postoperative outcome. We shall expand the number of cardiac centers in order to obtain comprehensive results of clinical outcomes for future research.

## 5. Conclusions

Both the CCI^®^ and CDC demonstrated a positive correlation with clinical outcomes, including the ICU stay, LOS, and hospitalization cost, while the CCI^®^ exhibited superior validity to the CDC in predicting the clinical outcomes of the patients who experienced cardiac surgery. Hence, the CCI^®^ serves as a valuable tool for predicting clinical outcomes following cardiac surgery, enabling healthcare teams to objectively and promptly evaluate the severity of complications, thereby facilitating earlier interventions and mitigating the incidence of severe complications and mortality.

## Figures and Tables

**Table 1:** General information of the subjects.

Item	n (%)	Median (P25, P75)
Gender		
Male	1121 (59.1)	
Female	775 (40.9)	
Age (Year)		58.0 (49.0, 65.0)
BMI		23.23 (21.08, 25.60)
Residence Type		
Urban	897 (47.3)	
Suburban	495 (26.1)	
Rural	504 (26.6)	
Marital Status		
Married	1563 (82.4)	
Unmarried	86 (4.5)	
Divorced	41 (2.2)	
Widowed	49 (2.6)	
Not Specified	157 (8.3)	
Comorbidities		
No Comorbidity	953 (50.3)	
Hypertension	382 (20.1)	
Diabetes	72 (3.8)	
Hypertension with Diabetes	96 (5.1)	
Stroke	65 (3.4)	
Hypertension with Stroke	26 (1.4)	
Hypertension with Diabetes and/or Stroke	12 (0.6)	
Renal Dysfunction	23 (1.2)	
Gastrointestinal Disease	251 (13.2)	
Others	124 (6.5)	
Resuscitation Records		
Type of Cardiac Surgeries		
Valve surgery	1115 (58.8)	
CABG surgery	210 (11.1)	
Valve + CABG surgery	48 (2.5)	
AD surgery	282 (14.9)	
Other types	241 (12.7)	
CCI^®^ Scores		
Total score		0.0 (0.0, 20.9)
For mild complications		20.9 (20.9, 29.6)
For severe complications		45.6 (37.1, 58.0)
CDC Grade		
0	1047 (55.2)	
Grade I	3 (0.2)	
Grade II	635 (33.5)	
Grade IIIa	104 (5.5)	
Grade IIIb	14 (0.7)	
Grade IVa	90 (4.7)	
Grade IVb	3 (0.2)	
No. of Postoperative Complications		
Single	362 (19.1)	
Two or more	487 (25.7)	
No. of CPB During the Surgery	1606 (84.7)	
Albumin Use	223 (11.8)	
Blood Products Use	223 (11.8)	
Surgery Time (min)		340 (289, 410)
CPB Time (min)		147 (108, 192)
ACC Time (min)		86 (57, 115)
ICU Stay (Days)		2.0 (1.0, 4.0)
LOS (Days)		14.0 (11.0, 18.0)
Hospital Cost (CNY) × 10^3^		116.5 (91.9, 160.2)

BMI: body mass index; CABG: coronary artery bypass grafting; AD: aortic dissection; CCI^®^: comprehensive complication index; CDC: Clavien–Dindo classification; CPB: cardiopulmonary bypass; ACC: aortic cross clamp; ICU: intensive care unit; LOS: length of hospital stay; CNY: Chinese Yuan.

**Table 2:** The quantity and percentage of postoperative complications in the subjects.

Complication	n (%)
Pneumonia	788 (92.8)
Auto-respiratory dysfunction	290 (34.2)
Arrhythmia	265 (31.2)
Heart failure	129 (15.2)
Anemia	78 (9.2)
Acute kidney injury	73 (8.6)
Postoperative neurocognitive disorders	66 (7.8)
Pleural effusion	63 (7.4)
Bleeding	61 (7.2)
Liver dysfunction	27 (3.2)
Cerebrovascular accident	22 (2.6)
Pulmonary atelectasis	21 (2.5)
Pneumothorax	19 (2.2)
Acute myocardial infarction	18 (2.1)
Cardiac arrest	12 (1.4)
Acute pancreatitis	9 (1.1)
Septicemia	6 (0.7)
Pericardial effusion	4 (0.5)
Surgical site infection (sternum)	4 (0.5)
Urinary tract infection	2 (0.2)
Intestinal obstruction	2 (0.2)
Mesentery ischemia	1 (0.1)

**Table 3:** Generalized linear models analysis of the variables associated with ICU stay/LOS/hospitalization cost.

Dependent Variable	$\hat{\beta }$ (95% CI)	*p*
ICU stay		
Intercept	0.502 (0.260–0.744)	<0.001
Age (year)	0.005 (0.003–0.007)	<0.001
Resuscitation record		
No	Ref	
Yes	0.480 (0.369–0.591)	<0.001
Surgery time (min)	0.001 (0.001–0.001)	<0.001
Albumin use		
No	Ref	
Yes	0.202 (0.110–0.295)	<0.001
CCI^®^ score	0.033 (0.031–0.034)	<0.001
BMI	0.011 (0.005–0.018)	0.001
Type of cardiac surgeries		
Valve surgery	Ref	
CABG surgery	−0.043 (−0.146–0.061)	0.421
Valve + CABG surgery	−0.059 (−0.208–0.093)	0.451
AD surgery	−0.063 (−0.147–0.022)	0.146
Other types	−0.102 (−0.183–−0.021)	0.015
LOS		
Intercept	1.915 (1.792–2.038)	<0.001
Age (year)	0.007 (0.005–0.008)	<0.001
Resuscitation record		
No	Ref	
Yes	0.277 (0.232–0.321)	<0.001
Surgery time (min)	0.001 (0.001–0.001)	<0.001
CCI^®^ score	0.010 (0.009–0.010)	<0.001
Comorbidities		
No	Ref	
Yes	0.058 (0.033–0.082)	<0.001
Type of cardiac surgeries		
Valve surgery	Ref	
CABG surgery	0.136 (0.085–0.186)	<0.001
Valve + CABG surgery	−0.040 (−0.110–0.028)	0.248
AD surgery	−0.032 (−0.072–0.009)	0.124
Other types	0.012 (−0.030–0.054)	0.576
Hospitalization cost		
Intercept	11.056 (10.890–11.223)	<0.001
Age (year)	0.008 (0.006–0.009)	<0.001
Resuscitation record		
No	Ref	
Yes	0.187 (0.111–0.264)	<0.001
Surgery time (min)	0.001 (0.001–0.002)	<0.001
Albumin use		
No	Ref	
Yes	0.224 (0.161–0.287)	<0.001
CCI^®^ score	0.011 (0.010–0.012)	<0.001
Type of cardiac surgeries		
Valve surgery	Ref	
CABG surgery	−0.410 (−0.479–−0.341)	<0.001
Valve + CABG surgery	−0.047 (−0.150–0.059)	0.380
AD surgery	0.026 (−0.031–0.084)	0.374
Other types	−0.082 (−0.139–−0.024)	0.004

Note: ICU stay and LOS using Poisson regression, hospitalization cost using Gamma regression; ICU: intensive care unit; LOS: length of hospital stay; CI: confidence interval; Ref: Reference; CCI^®^: comprehensive complication index; BMI: body mass index; CABG: coronary artery bypass grafting; AD: aortic dissection.

**Table 4:** The correlation coefficients (ρ) of CCI^®^ and CDC with clinical outcomes in the subjects.

Correlation Coefficients (ρ)	CCI^®^ (ρ)	*p*	CDC (ρ)	*p*	*p* CCI^®^ (ρ) &CDC (ρ)
ICU Stay	0.786	<0.001	0.401	<0.001	<0.001
LOS	0.465	<0.001	0.342	<0.001	0.002
Hospitalization Cost	0.602	<0.001	0.354	<0.001	<0.001

CCI^®^: comprehensive complication index; CDC: Clavien–Dindo classification; ICU: intensive care unit; LOS: length of hospital stays.

## Data Availability

The datasets from the current study are not publicly available due to patient privacy, but some relevant data can be obtained from the corresponding author on reasonable request.

## Supplementary Materials

The following supporting information can be downloaded at: https://www.mdpi.com/article/10.3390/jcdd12120461/s1, Table S1: Information for severity and intervention of postoperative complications; Supplementary file STROBE checklist.
