# Supplementary material for: Evaluation of the Comprehensive Complication Index Versus the Clavien–Dindo Classification for Predicting Clinical Outcomes After Cardiac Surgery in Adult Patients
Source: J Cardiovasc Dev Dis. 2025 Nov 27;12(12):461. doi: 10.3390/jcdd12120461 (PMC12733768; doi:10.3390/jcdd12120461)
Supplement: Supplementary file 1 [file jcdd-12-00461-s001.zip › Table S1.pdf]

Supplementary Table S1. Information for severity and intervention of postoperative complications

**Treatments applied to the cases with CDC graded complications**	**n**	**CCI® score**
I		8.7 (8.7, 8.7)*
Intubation for more than 48 hours	2	
Ischemic stroke identified by CT without intervention	1	
II		20.9 (20.9, 29.6)*
Vasodilator drugs for heart failure	37	
Anti-arrhythmia drugs	161	
Drugs for anti-platelet and decrease-cranial pressure for cerebrovascular accident	3	
Antibiotics upgraded for pneumonia	606	
Blood transfusion for anemia or bleeding	34	
Drugs for kidney protection	8	
Drugs for liver protection	12	
Drugs for PND	31	
Drugs for pancreatitis	2	
IIIa		38.4 (33.5, 40.5)*
Vasodilator drugs upgraded for heart failure	39	
Pericardial puncture for pericardial effusion	2	
Decompressive craniectomy for cerebrovascular accident	1	
Expectoration by laryngoscopy or bronchoscopy for pulmonary atelectasis	4	
Thoracentesis and drainage for pleural effusion or pneumothorax	55	
IIIb		44.1±5.9#
Open the chest again to stop the bleeding	13	
Sputum suction using tracheotomy for pulmonary atelectasis	1	
IVa		58.6 (51.7, 67.7)*
IABP for heart failure/AMI	16	
Electrical defibrillation/conversion for arrhythmia	36	
CPR for heart failure/arrhythmia	10	
Hemodialysis for AKI	38	
Plasma exchange for liver dysfunction	2	
Intravenous vasopressor drugs to treat septic shock for septicemia	4	
Delayed sternal closure	3	
IVb		99.0 (85.9, -)*
ECMO for heart failure/AMI	2	
MODS	1	

CT: computed tomography; PND: postoperative neurocognitive dysfunction; IABP: intra-aortic ballon pump; AMI: acute myocardial infarction; CPR: cardiopulmonary resuscitation; AKI: acute kidney injury; ECMO: extracorporeal membrane oxygenation; MODS: multiple organ dysfunction. #: mean±SD; *: median, IQR.
